# Supplementary material for: Risk Assessment of Indoor Air Quality and Its Association with Subjective Symptoms among Office Workers in Korea
Source: Int J Environ Res Public Health. 2022 Feb 20;19(4):2446. doi: 10.3390/ijerph19042446 (PMC8874417; doi:10.3390/ijerph19042446)
Supplement: Supplementary file 1 [file ijerph-19-02446-s001.zip › ijerph-1531859-supplementary.pdf]

Table S1. Summary of exposure factors, toxicity reference values, and distribution used in exposure scenarios for assessing the risk in offices.

**Exposure factor**	**Scenario value**	**Distribution**
Air concentration in office inhaled air (C)	Measurement value (μg/m^3)	Log-normal
Body weight (BW)	64.2±6.42 (kg)	Normal
Inhalation rate (IR)	14.3±1.43 (m3/day)	Normal
Exposure time (ET)	8.19±2.90 (hr/day)	Normal
Exposure duration (ED)	35 (year)	Uniform
Exposure frequency (EF)	250 (day/year)	Uniform
Average time (AT)	70 (year = 25,550 day/year)	Uniform

**Chemicals**		**SF (mg/kg/day)$^{-1}$**	**RfC (mg/m^3)**	**RfD (mg/kg/day)**
Carcinogens	Benzene	0.027	-	-
	Formaldehyde	0.045	-	-
	Acetaldehyde	0.0077	-	-
Non-carcinogens	Toluene	-	5.00	1.11
	Ethylbenzene	-	1.00	2.22×10^{-1}
	Xylene	-	1.00×10^{-1}	2.23×10^{-2}
	NO_2	-	12.3×10^{-2}	0.027
	O_3	-	12.9×10^{-2}	0.029

Table S2. Characteristics of indoor office environments (N=31).

**Category**		**Mean (S.D) / N (%)**
Number of workers		27.94 (28.76)
Working hours (hr/day)		8.19 (2.90)
Office volume (m^3)		674.52 (743.82)
Number of computers		24.23 (27.09)
Number of printers		3.23 (4.86)
Number of photocopiers		4.00 (5.65)
Frequency of ventilation		1.92 (2.56)
Average ventilation duration (min/unit)		27.17 (42.51)
Average time spent using air purifier (hr/day)		7.07 (5.28)
Average time spent using ventilation (hr/day)		8.30 (7.84)
Charcoal or plant pot	Yes	24 (77.34)
	No	7 (22.6)
Gas range	Yes	1 (2.9)
	No	30 (96.8)
Ventilator	Yes	14 (45.2)
	No	17 (54.8)
Air purifier	Yes	9 (29)
	No	22 (71)
HVAC	Yes	15 (48.4)
	No	16 (51.6)
HVAC operational	Yes	3 (9.7)
	No	12 (38.7)
HVAC inspection	Yes	1 (6.7)
	No	14 (93.3)

* HAVC : Heating, ventilation, and air conditioning.

Table S3. Characteristics according to three categories offices.

Category	Group 1* (N=10)	Group 2** (N=10)	Group 3*** (N=11)
		Mean (SD) and range (min-max)	
Number of workers	28.80 (19.83), 7.00-65.00	23.70 (21.01), 4.00-70.00	31.00 (41.25), 3.00-150.00
Floor area (m^2)	190.01 (137.76), 28.53-510.40	236.54 (126.55), 66.06-394.02)	339.91 (445.17), 27.69-1614.17
Office volume (m^3)	502.61 (379.05), 69.82-1398.50	604.17 (327.76), 155.86-1063.02	894.77 (1160.16), 98.84-4196.32
Distance of car street (m)	62.60 (93.21), 0.00-300.00	161.50 (147.61), 5.00-500.00	417.00 (351.82), 1000.00-20.00

*Group 1 (general office in metropolitan city): university, bank, broadcasting station, insurance company, business building, bus company, hospital, governmental institution

**Group 2 (general office in Ulsan): governmental institution, business building, hospital

***Group 3 (detached office from factory within company site boundary): company manufacturing display machine, automobile and machine equipment, printing paper, household items, automobile interior material, automobile engine, building material, industrial rubber.

Table S4. General environment characteristics for three office groups

Category		Construction year	Volume (m^3)	Floor area (m^2)	Workers (n)	Computer (n)	Photocopier (n)	Windows (n)	Distance from car street (m)	Existence of HVAC
Group 1	University (1)	2004	344.84	132.63	7	8	2	0	100	No
	University (2)	2002	222.10	88.17	12	12	3	4	100	Yes
	Bank	1985	766.14	310.18	36	35	2	19	5	Yes
	Broadcasting station	1994	1398.50	510.40	40	19	3	9	5	No
	Insurance company	2010	679.12	227.65	50	58	1	20	5	No
	Business building	2016	215.08	82.38	8	8	1	8	30	No
	Bus company	2000	69.82	28.53	65	1	1	2	80	No
	Hospital (1)	2005	488.62	182.32	21	23	18	0	-	Yes
	Hospital (2)	1950	383.61	164.92	37	34	18	20	1	No
	Governmental institution	2011	458.24	172.92	12	12	1	19	300	Yes
Group 2	Governmental institution (1)	2012	904.06	335.09	28	28	15	21	200	No
	Governmental institution (2)	2016	336.79	122.03	4	4	1	16	10	No
	Governmental institution (3)	2014	563.68	195.25	20	19	5	14	200	Yes
	Governmental institution (4)	2005	175.33	66.06	6	6	1	8	20	Yes
	Governmental institution (5)	2009	474.09	140.01	11	13	1	1	500	No
	Governmental institution (6)	2014	628.38	242.62	21	21	3	11	200	Yes
	Business building (1)	2016	1063.02	386.01	49	49	5	34	200	Yes
	Business building (2)	2016	713.28	111.80	7	15	0	0	200	Yes
	Business building (3)	2013	1027.20	394.02	70	31	2	101	80	Yes
	Hospital	1999	155.86	372.51	21	22	2	18	5	Yes
Group 3	Company manufacturing display machine	2004	4196.32	1614.17	150	150	2	14	20	Yes
	Company manufacturing automobile and machine equipment	2016	447.53	157.60	15	8	1	24	100	No
	Company manufacturing printing paper	2016	1214.15	446.38	42	38	3	8	700	No
	Company manufacturing household items (1)	1975	1317.11	474.00	36	36	2	6	1000	No
	Company manufacturing household items (2)	1995	229.47	86.95	6	6	3	5	400	Yes
	Company manufacturing automobile interior material	1985	607.06	252.85	9	9	1	10	50	No
	Company manufacturing automobile engine (1)	1981	414.64	162.22	19	19	1	14	-	No
	Company manufacturing automobile engine (2)	1977	415.76	153.42	19	19	3	19	900	Yes
	Company manufacturing building material	2004	98.84	27.69	3	1	1	6	300	No
	Company manufacturing industrial rubber	1995	269.32	110.92	15	20	21	4	200	Yes
	Company manufacturing building and interior material	2000	632.24	252.79	27	27	1	11	500	No

Table S5. Air pollutant concentrations measured in indoor and outdoor office environments.

	Location	N	Detection rate (%)	M±SD	GM	50%	95%	I/O
Temperature (°C)	Indoor	31	100	26.74±2.38	26.64	26.30	32.02	0.94
	Outdoor	31	100	28.58±3.55	28.38	28.10	36.18	
Humidity (%)	Indoor	31	100	56.94±11.51	55.69	58.8	77.02	0.86
	Outdoor	31	100	66.26±11.06	65.33	67.9	87.66	
CO (ppm)	Indoor	13	41.94	0.35±0.43	0.16	0.17	N/D	0.90
	Outdoor	13	41.94	0.39±0.41	0.17	0.23	N/D	
CO_2 (ppm)	Indoor	31	100	757.84±303.82	709.52	728.67	1518.33	1.73
	Outdoor	31	100	439.03±68.94	434.03	422.83	590.67	
O_3 (ppm)	Indoor	22	70.97	0.03±0.01	0.02	0.03	0.05	0.75
	Outdoor	31	100	0.04±0.02	0.03	0.03	0.08	
NO_2 (ppm)	Indoor	25	80.65	0.03±0.01	0.02	0.02	0.06	1.00
	Outdoor	25	80.65	0.03±0.02	0.03	0.03	0.08	
HCHO (μg/m^3)	Indoor	31	100	40.93±49.26	20.73	32.23	176.00	6.24
	Outdoor	31	100	6.56±3.36	5.31	7.19	11.51	
TVOCs (μg/m^3)	Indoor	31	100	358.07±280.52	274.29	235.83	1066.58	1.91
	Outdoor	31	100	187.47±105.96	156.89	165.11	410.32	
Benzene (μg/m^3)	Indoor	31	100	4.13±3.91	2.92	1.99	12.50	1.51
	Outdoor	31	100	2.73±3.19	1.90	1.49	8.01	
Toluene (μg/m^3)	Indoor	31	100	121.33±125.36	87.00	83.98	265.30	1.84
	Outdoor	31	100	66.02±55.54	39.32	46.40	135.87	
Ethylbenzene (μg/m^3)	Indoor	31	100	13.52±9.40	10.29	13.09	26.36	1.36
	Outdoor	31	100	9.95±9.34	6.62	6.83	31.05	
m,p-Xylene (μg/m^3)	Indoor	31	100	9.15±5.86	7.34	9.14	19.94	1.16
	Outdoor	31	100	7.88±6.28	6.62	6.12	18.72	
o-Xylene (μg/m^3)	Indoor	31	100	6.32±4.02	5.08	6.43	13.19	1.16
	Outdoor	31	100	5.44±4.06	4.04	4.90	12.39	
Acetaldehyde (μg/m^3)	Indoor	31	100	13.62±10.64	9.83	11.56	31.40	2.70
	Outdoor	31	100	5.05±2.38	4.26	5.31	8.59	

Table S6. Air pollutants concentration according to three classifications

Pollutant	Category	Indoor			Outdoor		
		N	M ± SD	p-value	N	M ± SD	p-value
Temperature (℃)	Group 1	10	26.29 ± 2.25	0.43	10	28.31 ± 2.34	0.22
	Group 2	10	26.67 ± 2.97		10	30.19 ± 4.51	
	Group 3	11	25.95 ± 1.71		11	27.36 ± 3.22	
Humidity (%)	Group 1	10	60.83 ± 5.31	0.54	10	71.30 ± 8.05	0.12
	Group 2	10	55.14 ± 13.49		10	66.74 ± 12.74	
	Group 3	11	55.03 ± 13.65		11	61.25 ± 10.48	
CO_2 (ppm)	Group 1	10	778.62 ± 218.43	0.10	10	470.87 ± 70.18	0.13
	Group 2	10	883.28 ± 413.17		10	427.72 ± 67.81	
	Group 3	11	624.92 ± 211.51		11	420.38 ± 64.52	
O_3 (ppm)	Group 1	9	0.03 ± 0.02	0.75	10	0.04 ± 0.03	0.45
	Group 2	7	0.03 ± 0.01		10	0.04 ± 0.02	
	Group 3	9	0.03 ± 0.01		9	0.03 ± 0.02	
NO_2 (ppm)	Group 1	9	0.03 ± 0.01	0.26	9	0.03 ± 0.02	0.63
	Group 2	7	0.02 ± 0.02		7	0.03 ± 0.02	
	Group 3	9	0.03 ± 0.01		9	0.03 ± 0.02	
HCHO (μg/m^3)	Group 1	10	32.12 ± 20.83	0.13	10	7.62 ± 2.66	0.43
	Group 2	10	69.06 ± 76.34		10	6.41 ± 3.77	
	Group 3	11	23.37 ± 20.93		11	5.71 ± 3.55	
TVOCs (μg/m^3)	Group 1	10	364.20 ± 266.04	0.46	10	169.11 ± 100.31	0.55
	Group 2	10	334.93 ± 144.34		10	226.44 ± 121.91	
	Group 3	11	373.54 ± 390.89		11	168.75 ± 94.93	
Benzene (μg/m^3)	Group 1	10	4.48 ± 4.31	0.048	10	3.16 ± 4.50	0.65
	Group 2	10	5.85 ± 3.72		10	3.39 ± 3.33	
	Group 3	11	2.82 ± 4.03		10	2.36 ± 2.44	
Toluene (μg/m^3)	Group 1	10	146.37 ± 88.48	0.13	10	39.41 ± 43.03	0.14
	Group 2	10	108.11 ± 63.32		10	87.13 ± 69.38	
	Group 3	10	125.44 ± 193.67		10	56.25 ± 42.21	
Ethylbenzene (μg/m^3)	Group 1	10	16.20 ± 8.34	0.057	10	8.51 ± 7.67	0.73
	Group 2	10	15.92 ± 7.53		10	12.00 ± 9.82	
	Group 3	10	9.86 ± 11.58		10	10.23 ± 10.85	
m,p-Xylene (μg/m^3)	Group 1	10	10.75 ± 6.14	0.14	10	7.48 ± 6.44	0.53
	Group 2	10	10.70 ± 5.34		10	10.21 ± 7.45	
	Group 3	10	7.11 ± 6.53		10	6.87 ± 5.39	
o-Xylene (μg/m^3)	Group 1	10	6.67 ± 4.54	0.26	10	5.31 ± 4.23	0.62
	Group 2	10	7.58 ± 3.34		10	6.68 ± 4.66	
	Group 3	10	5.36 ± 4.53		10	4.89 ± 3.67	
Acetaldehyde (μg/m^3)	Group 1	10	10.36 ± 5.53	0.06	10	6.30 ± 2.19	0.11
	Group 2	10	21.89 ± 14.01		10	4.33 ± 2.02	
	Group 3	10	8.97 ± 6.10		10	4.96 ± 2.37	

Table S7. Correlation among indoor air pollutant concentrations measured in office buildings (shaded areas represent outdoor concentrations).

	(1)	(2)	(3)	(4)	(5)	(6)	(7)	(8)	(9)	(10)	(11)	(12)	(13)	(14)
(1)	1	.027	-.539	.075	.221	-.174	.489**	.420*	.046	.000	-.031	.013	.024	-.024
	.014	.884	.057	.688	.239	.405	**.005**	**.019**	.806	.999	.870	.944	.896	.898
(2)	-.436*	1	.676*	.165	-.097	.215	-.131	.231	-.189	.100	.147	.027	-.045	-.090
	**.052**	.059	**.011**	.376	.611	.303	.483	.211	.308	.593	.430	.886	.810	.629
(3)	-.205	.202	1	.405	-.420	.330	-.449	-.122	.199	.011	-.153	.053	.082	.064
	.417	.402	.488	.170	.153	.294	.124	.692	.514	.971	.618	.864	.791	.834
(4)	.151	-.156	.211	1	.414*	.100	-.103	-.499**	-.297	-.101	-.049	-.102	-.089	-.128
	.526	.391	.205	.612	**.023**	.635	.581	**.004**	.105	.590	.794	.587	.634	.491
(5)	.147	-.197	-.052	.118	1	-.519**	.126	-.128	-.100	.077	-.015	.043	-.029	-.069
	.526	.391	.205	.612		**.009**	.507	.499	.600	.687	.938	.822	.880	.718
(6)	-.233	.399*	.562	-.147	-.595*	1	-.099	.078	-.105	-.109	-.122	-.092	.057	.093
	.263	**.048**	.057	.484	**.019**		.639	.709	.616	.603	.560	.662	.787	.658
(7)	.196	-.110	-.261	.290	.375	-.298	1	.157	-.164	.103	.033	.274	.197	.170
	.292	.556	.388	.113	.093	.148		.398	.377	.580	.859	.137	.289	.361
(8)	.098	.282	.066	.055	-.091	-.294	-.107	1	.042	.135	.070	-.112	-.055	-.043
	.600	.125	.830	.770	.696	.153	.568		.821	.468	.708	.548	.771	.820
(9)	.120	.220	.244	-.021	-.072	.033	-.010	.134	1	.091	.005	.054	.042	.074
	.522	.235	.422	.912	.756	.875	.959	.472		.626	.980	.773	.821	.692
(10)	.270	.277	-.480	.053	.147	-.327	.121	.287	.067	1	.169	.461**	.332	.339
	.141	.132	.097	.777	.524	.111	.517	.118	.721		.363	**.009**	.068	.062
(11)	.065	.112	.090	.328	.260	.004	-.027	.214	-.161	.409*	1	.513**	.534**	.500**
	.730	.550	.770	.072	.254	.984	.885	.248	.388	**.022**		**.003**	**.002**	**.004**
(12)	.104	.138	-.010	.219	.147	-.283	.148	.284	.177	.617**	.686**	1	.937**	.915**
	.576	.460	.974	.237	.526	.170	.427	.122	.340	**.000**	**.000**		**.000**	**.000**
(13)	.089	.090	-.126	.122	.106	-.277	.176	.217	.179	.586**	.625**	.970**	1	.991**
	.633	.629	.681	.512	.648	.181	.344	.241	.337	**.001**	**.000**	**.000**		**.000**
(14)	.095	.105	-.104	.214	-.027	-.257	.240	.169	.113	.568**	.551**	.835**	.849**	1
	.611	.573	.735	.248	.907	.214	.193	.364	.544	**.001**	**.001**	**.000**	**.000**	

(1) Temperature (2) Humidity (3) CO (4) CO_2 (5) O_3 (6) NO_2 (7) TVOCs (8) HCHO (9) Acetaldehyde (10) Benzene (11) Toluene (12) Ethylbenzene (13) m,p-Xylene (14) o-Xylene

*Correlation is significant at the 0.01 level
**Correlation is significant at the 0.05 level
